# New horizons in the pathogenesis, diagnosis and management of sarcopenia

**DOI:** 10.1093/ageing/afs191

**Published:** 2013-01-11

**Authors:** Avan Aihie Sayer, Sian M. Robinson, Harnish P. Patel, Tea Shavlakadze, Cyrus Cooper, Miranda D. Grounds

**Affiliations:** 1 Academic Geriatric Medicine, University of Southampton, Southampton, UK; 2 MRC Lifecourse Epidemiology Unit, University of Southampton, Southampton, UK; 3 School of Anatomy, Physiology and Human Biology, The University of Western Australia, Western Australia, Australia

**Keywords:** sarcopenia, skeletal muscle, ageing, pathogenesis, diagnosis, exercise, nutrition, drug treatment, life course, epidemiology, older people

## Abstract

Sarcopenia is the age-related loss of skeletal muscle mass and function. It is now recognised as a major clinical problem for older people and research in the area is expanding exponentially. One of the most important recent developments has been convergence in the operational definition of sarcopenia combining measures of muscle mass and strength or physical performance. This has been accompanied by considerable progress in understanding of pathogenesis from animal models of sarcopenia. Well-described risk factors include age, gender and levels of physical activity and this knowledge is now being translated into effective management strategies including resistance exercise with recent interest in the additional role of nutritional intervention. Sarcopenia is currently a major focus for drug discovery and development although there remains debate about the best primary outcome measure for trials, and various promising avenues to date have proved unsatisfactory. The concept of ‘new tricks for old drugs’ is, however, promising, for example, there is some evidence that the angiotensin-converting enzyme inhibitors may improve physical performance. Future directions will include a deeper understanding of the molecular and cellular mechanisms of sarcopenia and the application of a lifecourse approach to understanding aetiology as well as to informing the optimal timing of interventions.

## Introduction

Sarcopenia, the age-related loss of skeletal muscle mass and function, is coming of age. It is now recognised as a major clinical problem for older people and research in the area is expanding exponentially [1]. This interest stems from the fact that sarcopenia is both common and associated with serious health consequences in terms of frailty, disability, morbidity and mortality. The estimated direct healthcare cost attributable to sarcopenia in the USA in 2000 was £18.5 bn [2]. Furthermore, sarcopenia is associated with major co-morbidity such as obesity, osteoporosis and type 2 diabetes [3]. But perhaps the most powerful indication that the loss of skeletal muscle, in particular strength, is important comes from the evidence that it predicts future mortality in middle-aged as well as older adults [4].

One of the most important recent developments has been convergence in the operational definition of sarcopenia. The European Working Group on Sarcopenia in Older People published guidelines in 2010 where specific parameters to identify sarcopenia have been identified [5]. Similar (although not identical) guidelines have subsequently emerged from the USA [6]. This step forward in defining sarcopenia is leading to opportunities in two major areas: first in understanding aetiology and secondly in developing treatments. This progress is underpinned by studies in animal (especially rodent) models of sarcopenia [7]. These provide the opportunity for intensive experimentation to define the molecular and cellular changes that lead to, and are the hallmarks of, this age-related condition.

Well-described risk factors for sarcopenia include age, gender and level of physical activity, and resistance exercise is particularly effective for slowing the age-related loss of skeletal muscle. Nevertheless many gaps in our knowledge remain and there are a number of promising new horizons. This review will focus on current understanding as well as new developments in the pathogenesis, diagnosis and management of sarcopenia.

## Pathogenesis

Skeletal muscle comprises primarily two main types of muscle fibre (myofibre): type 1 myofibres have a slow contraction time, utilise oxidative pathways and resist fatigue. In contrast, type 2 myofibres have a quick contraction time, rely on glycolytic pathways and fatigue more easily. The age-related loss of human skeletal muscle mass is due to a decrease in myofibre size and number with the loss of both fast and slow type myofibres, although the loss of fast myofibres tends to start earlier, at ∼70 years [8]. Many factors influence the decrease in muscle mass. A significant contributor is an anabolic resistance of older skeletal muscle to protein nutrition [9] as seen during immobilisation [10] which can be ameliorated at least in part by resistance exercise and dietary supplementation [11]. Other intensive areas of research are related to the loss of innervation [12] and oxidative damage [13]. Loss of myofibre innervation is a characteristic of ageing muscles with changes occurring at many levels, from the central and peripheral nervous system to cells in skeletal muscle tissue. These include the loss of motoneurons in the central nervous system (CNS), diminished function of the remaining motoneurons, demyelination of axons and withdrawal of nerve terminals from the neuromuscular junctions (NMJs) [12, 14, 15].

**Figure 1.**
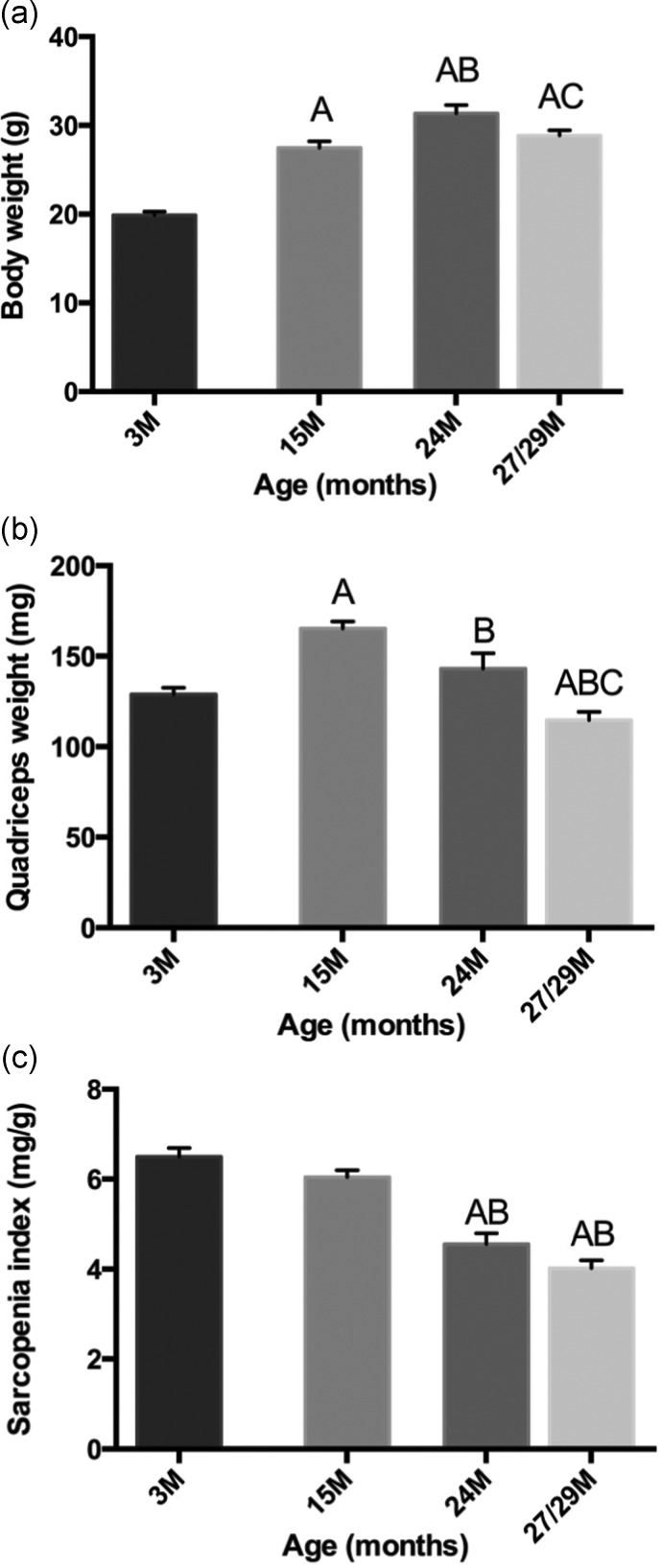
Mice as a model for sarcopenia: changes in (a) body mass and (b) weight of quadriceps muscles over the life-span of female C57Bl/6J mice expressed as (c) a sarcopenia index [16]. The loss of quadriceps muscle mass starts sometime after 15 months of age, is evident by 24 months and pronounced by 29 months. Statistically significant differences between ages (*P* < 0.05) are shown as A: different from 3 months, B: different from 15 months and C: different from 24 months. The extent of sarcopenia is influenced by gender (being more pronounced in females than males) and may vary between different muscles.

Many studies that describe the pathogenesis of ageing skeletal muscle have been carried out in rodent models. In contrast with human muscles that are composed predominantly of slow myofibres, mouse muscles are mainly fast: such differences between species need to be considered when extending observation from animal models to humans. Furthermore, there is more time for more secondary consequences to become pronounced in humans where sarcopenia becomes progressively manifest over 20–30 years, whereas the duration is far shorter in mice; >1 year (from ∼18 to 30 months), with the normal lifespan of mice being a mere 3 years or less (Figure 1). Innervation of myofibres is clearly required for skeletal muscle contraction in mice and humans, but different conclusions may be reached from initial studies. Examination of aged mice (up to 29 months old) revealed marked denervation of NMJs of hind limb muscles without any change in number or size of motorneuron cell bodies in the lumbar spinal cord, suggesting a primary problem at the level of the muscles *per se* [16]. In contrast, many changes in motorneurone function are noted from electrophysiological studies in ageing humans supporting changes in the CNS [15], although it is difficult to determine whether these changes are secondary to earlier NMJ changes, since invasive examination of the NMJ status is rare in human studies. Further experiments in animal models can help to define the precise timing of these key events.

In older age, an accumulation of reactive oxygen species (ROS) may lead to oxidative damage of biomolecules, and contribute to the loss of muscle mass and strength. It is well documented that elevated oxidative stress is associated with many clinical situations of muscle wasting, but the precise nature of the oxidative stress in different situations and their complex *in vivo* interactions remain unclear [17]. Irreversible oxidation of macromolecules such as proteins and lipids results in the accumulation of a pigment called lipofuscin that is a classic marker for ageing tissues [18]. Other forms of ROS cause reversible oxidation of protein thiols to modulate the function of many proteins (e.g. involved in signalling regulating protein synthesis and degradation, muscle contraction), although the contribution of such thiol oxidation to sarcopenia has barely been evaluated [17]. Since different anti-oxidants target specific types of ROS, it is essential to know exactly what forms of ROS are elevated in sarcopenia in order to select the appropriate therapeutic drug or supplement.

## Diagnosis

**Figure 2.**
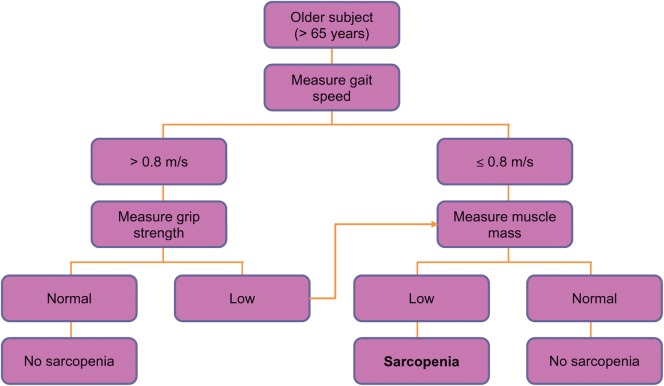
EWGSOP-suggested algorithm for sarcopenia case finding in older individuals [5].

The European Working Group on Sarcopenia in Older People (EWGSOP) have recently developed a practical clinical definition as well as consensus criteria for sarcopenia allowing an important step forward in the field in terms of standardising diagnosis [5]. The recommendation is to use the presence of both low muscle mass and low muscle function (strength or performance) as summarised in an algorithm (Figure 2). The approach applies these characteristics to further define conceptual stages as presarcopenia, sarcopenia and severe sarcopenia.

Encouragingly there seems to be international convergence in the approach to defining sarcopenia. Fielding and colleagues published US guidelines last year on behalf of the International Working Group on Sarcopenia suggesting that a diagnosis of sarcopenia could be made on the basis of low gait speed and an objectively measured low muscle mass [6]. This area is important because an operational definition is needed to allow development and evaluation of interventions for prevention or treatment and with the emergence of a number of candidate therapies, this has become more pressing [19].

## Management

There is considerable interest in the role of lifestyle and diet in the aetiology of sarcopenia, and the extent to which interventions to change behaviour could make useful contributions to its management [20].

### Exercise

The established link between inactivity and losses of muscle mass and strength suggests that physical activity should be protective for sarcopenia. A range of exercise interventions, that include resistance, power and functional training, as well as endurance and aerobic training regimes, have been used. In particular, progressive resistance exercise training (PRT), in which participants exercise against an increasing external load, has been shown to have positive effects on strength and physical function. A 2009 Cochrane review of 121 randomised controlled trials showed PRT to be an effective intervention for improving physical functioning in older people [21]; observed benefits included large positive effects on muscle strength as well as improved performance in some assessed activities. Most commonly the exercise interventions were carried out two to three times per week. Strength training appears to evoke not only muscle hypertrophy, but also beneficial changes in neuromuscular function [15] and other types of exercise interventions, involving gait, balance, co-ordination and functional exercises, may also be effective in reducing the risk and rate of falls [22] as well as improving balance in older people [23].

### Diet and nutrition

In comparison with the clear benefits of exercise, we know much less about the influence of diet in older age on muscle mass and strength, and much of the research in this area is relatively new. Food intake falls by ∼25% between 40 and 70 years of age, putting older people at risk of having inadequate nutrient intakes. There is a growing literature that suggests that diet could be an important modifiable influence on sarcopenia [24]—with the most consistent evidence pointing to roles for protein, vitamin D and antioxidant nutrients.

Dietary protein provides amino acids needed for the synthesis of muscle protein, and absorbed amino acids have a stimulatory effect on protein synthesis after feeding. Branched chain amino acids, such as leucine, have been shown to boost signalling pathways that lead to increased protein translation in both humans and rodents [25, 26]. However, there is concern that these anabolic responses may be blunted in older people [27], raising the possibility that recommendations for protein intake should be increased [28]. There is good observational evidence that links low-protein intake to declining muscle mass [29], and supplementation with protein and/or amino acids should therefore have the potential to slow sarcopenic muscle loss. However, the results from trials have been inconsistent. A Cochrane review found that the use of protein and energy supplements in older people at risk of malnutrition produced a small but consistent weight gain and mortality appeared to be reduced in those who were undernourished [30]. However, there was no evidence of functional benefit and further work is needed to establish protein and specific amino acid requirements to support optimal physical function in older people.

The current widespread interest in the contribution of low vitamin D status to poor health also extends to effects on muscle strength and physical function in older adults. The vitamin D receptor (VDR) has been isolated from skeletal muscle, and polymorphisms of the VDR have been linked to differences in muscle strength [31]. Much of the observational literature is consistent with direct effects of vitamin D on muscle function. For example, a fourfold increase in the risk of frailty has been described in older men and women with low vitamin D status [32] and a meta-analysis indicated that vitamin D supplementation (700–1000 IU/day) reduces risk of falls in older people [33]. However, supplementation is not consistently linked to measurable improvements in physical function, and its benefits remain controversial [34]. Nevertheless as vitamin D insufficiency is common among older adults [32], further trial data are needed.

The implicated role of oxidative stress in the aetiology of sarcopenia has focused interest on the effects of dietary antioxidants [35]. Observational studies have shown better physical function among older adults with a higher antioxidant status and, importantly, low status is predictive of a decline in measures of function such as walking speed over time [36]. To date there have been few studies to determine how antioxidant supplementation of older adults affects muscle strength, and the benefits are uncertain. Interventions based on simple suppression of activity of ROS, through the use of non-specific antioxidants, may be unlikely to improve age-related declines in muscle mass and function [37], but this remains an important question to be addressed.

Much of the existing evidence that links diet to muscle mass and strength in older people is observational. Because dietary components are often highly correlated with each other, identifying the effects of individual nutrients is problematic. For example, high fruit and vegetable consumption may be indicators of other dietary differences which could be important for muscle function, such as greater intakes of vitamin D and antioxidant nutrients [38]. There is some evidence that ‘healthy’ diets, characterised by greater fruit and vegetable consumption and wholemeal cereals, are associated with greater muscle strength in older adults [24]. Interventions to change dietary patterns would be expected to change intakes of a range of nutrients, and therefore could be more effective than single-nutrient supplements in preventing age-related losses in muscle mass and strength. Despite the challenges of changing dietary behaviour, nutrition interventions in older community-dwelling adults have been shown to be effective [39]. The potential of whole-diet interventions for the management of sarcopenia is significant, and needs to be explored.

### Diet and exercise

A final consideration for maintenance of healthy muscle is the possibility of interactions between diet and exercise in their influence on muscle mass and strength, and the extent to which interventions that combine supplementation and exercise training may be more effective than either alone. Interactive effects of diet and exercise on physical function have been studied most extensively in relation to protein/amino acid supplementation. Although synergistic effects of protein feeding and exercise have been described [40], it is not clear whether there are additional benefits of protein/amino acid supplementation on the skeletal muscle response to prolonged resistance exercise training [41]. A recent report showed that initial benefits in older subjects were blunted over time [9]. At present the implications for long-term effects of combined exercise training and high-protein intakes for management of sarcopenia are uncertain. Further research is needed into the interactions between nutrition, exercise and skeletal muscle adaptations in order to define effective strategies to prevent and treat sarcopenia in the future [41].

### Drug treatment

Sarcopenia is now a major focus for drug discovery and development [42]. However, debate about the best primary outcome measure for trials is ongoing and various promising therapeutic avenues to date have proved unsatisfactory. Growth hormone has been used for some years to improve muscle mass, but the data supporting an associated increase in function have not been convincing [43]. The therapeutic use of testosterone had a setback recently when a major trial had to be stopped early because of side effects [44]. Perhaps a more promising area at present is the idea of ‘new tricks for old drugs’. For example, there is some evidence for the benefit of angiotensin-converting enzyme inhibitors in improving physical function which may be mediated through a direct effect on muscle, and a number of studies are currently underway in this area [45]. An important consideration that has previously often been overlooked is the potential for commonly used drugs to also have adverse effects on skeletal muscle function. For example, we have shown that many prescribed medications are associated with lower grip strength and this appears to be partly independent of co-morbidities [46].

## Future directions

**Figure 3.**
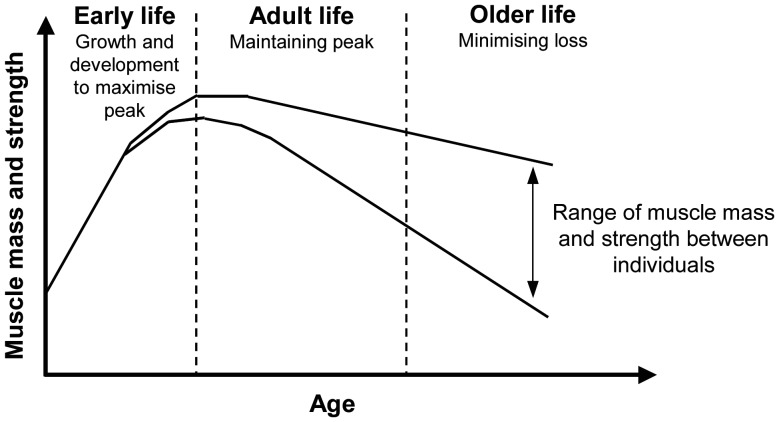
A lifecourse approach to sarcopenia [47].

A deeper understanding of the molecular and cellular mechanisms of sarcopenia, derived from both human and animal studies, has great potential to identify novel targets for drug and other treatment strategies as well as to develop better biomarkers to monitor the efficacy of various interventions. Another exciting new area is the application of a lifecourse approach to the understanding and management of sarcopenia. This approach recognises that muscle mass and function in later life reflect not only the rate of muscle loss, but also the peak attained earlier in life (Figure 3) [47]. Therefore, in addition to identifying the determinants of skeletal muscle loss, there needs to be focus on the factors associated with peak muscle mass and strength such as birth weight [48] and early nutrition [49] as well as the mechanisms underlying these associations [50]. Importantly, the lifecourse approach suggests that there is potential for prevention and intervention at earlier stages of life rather than just when sarcopenia has become established although to date the evidence is scanty. This is a fertile area for future research.

## Conflicts of interest

None declared.
